# Lessons From APOL1 Animal Models

**DOI:** 10.3389/fmed.2021.762901

**Published:** 2021-10-26

**Authors:** Teruhiko Yoshida, Khun Zaw Latt, Jurgen Heymann, Jeffrey B. Kopp

**Affiliations:** Kidney Disease Section, Kidney Diseases Branch, National Institute of Diabetes and Digestive and Kidney Diseases, National Institutes of Health (NIH), Bethesda, MD, United States

**Keywords:** APOL1, animal model, CKD—chronic kidney disease, glomerular diseases, podocyte

## Abstract

African-Americans have a three-fold higher rate of chronic kidney disease compared to European-Americans. Much of this excess risk is attributed to genetic variants in *APOL1*, encoding apolipoprotein L1, that are present only in individuals with sub-Saharan ancestry. Although 10 years have passed since the discovery of *APOL1* renal risk variants, the mechanisms by which *APOL1* risk allele gene products damage glomerular cells remain incompletely understood. Many mechanisms have been reported in cell culture models, but few have been demonstrated to be active in transgenic models. In this narrative review, we will review existing *APOL1* transgenic models, from flies to fish to mice; discuss findings and limitations from studies; and consider future research directions.

## Introduction

*APOL1* genetic variants are an important cause of kidney disease, affecting individuals who have sub-Saharan African ancestry (1). Apolipoprotein L1 is a component of the innate immune system. It is produced primarily by the liver but also by brain, kidney, and other tissues. APOL1 is a component of HDL particles, which serve as delivery platforms for multiple proteins related to defense against infectious disease. When African trypanosomes circulating in blood ingest HDL particles, APOL1 is released from the HDL particles and trafficked to the lysosomes where it forms ion channels, thereby killing the parasite and thus preventing African sleeping sickness.

Trypanosomes developed a defense protein, serum resistance antigen (SRA), that binds APOL1 and prevents trypanocide (2). In response to the evolutionary pressure, human *APOL1* genetic variants, APOL1-G1 and APOL1-G2, arose that interfere with SRA binding. The APOL1 variants thereby restore trypanosomal killing but do so at the cost of increased risk for chronic kidney disease. This parallels the relationship between malaria and sickle cell hemoglobin, whereby the latter protects to some extent against malaria, particularly severe malaria, and consequently has been evolutionarily selected for in Mediterranean and sub-Saharan Africa regions (3). However, this protection comes at the cost of a disabling disorder, sickle cell anemia. While the molecular mechanism leading to sickle cell anemia is well understood, this has not been the case for the involvement of APOL1 in glomerular injury.

Histologic manifestations of APOL1 kidney disease including focal segmental glomerulosclerosis, collapsing glomerulopathy, arterionephrosclerosis (also termed hypertension-associated kidney disease), and advanced forms of lupus nephritis (4–6). Other manifestations of APOL1 kidney disease include shorter renal allograft survival among recipients with two *APOL1* risk alleles and a rare but concerning gradual loss of kidney function among living kidney donors (7, 8). These risks are generally confined to those who carry two *APOL1* renal risk alleles (high risk genotype). The one setting where there is an increased risk associated with carriage of one *APOL1* risk allele is HIV-associated collapsing glomerulopathy in South Africa (9).

Current therapies for chronic kidney disease are less effective at preventing progressive loss of kidney function in individuals with *APOL1* high risk genotype, compared to other forms of glomerular disease, even when there may be a reduction in proteinuria in a particular patient. APOL1 may not be an essential protein, as one individual who is genetically null for *APOL1* appears phenotypically normal (10). However, it is only one case report and we obviously need further confirmation to understand function and necessity of APOL1. Further, APOL1 is unique to humans, gorillas, and baboons (11). Interestingly, the gene is absent from the genome of chimpanzees and bonobos, our nearest neighbors in evolutionary terms; this remains a puzzle. Taken together, these observations suggest that APOL1-targeted therapy for APOL1-associated kidney disease may be well-tolerated, except in regions with endemic African trypanosomiasis. One such approach is to suppress APOL1 protein levels; this is now investigated in clinical trials (12, 13). Other therapeutic approaches are also needed, and experimental animal models will be of great utility in testing the efficacy of such therapies, as well as improving understanding of APOL1 disease pathogenesis.

Criteria that are frequently applied when animal models are considered in the study of human disease include similarity of the model to humans with respect to physiology, anatomy and genetics. But others such as the level at which a model system can be manipulated, controlled, and examined and the scalability so to achieve statistical power are equally important.

## Mice

Mouse models are the most frequently used animal models and have been widely explored to investigate APOL1 kidney disease, even though mice do not have an ortholog to the human *APOL1* gene. The mouse apolipoprotein L family includes 12 genes and one pseudogene (*Apol6, Apol7a, Apol7b, Apol7c, Apol7e, Apol8, Apol9a, Apol9b, Apol10a, Apol10b, Apol11a, Apol11b*, and pseudogene *Apol10c*) clustered on chromosome 15 (14).

A primary aim of the *APOL1* transgenic mouse studies has been to characterize the molecular mechanism of kidney damage induced by *APOL1* renal risk alleles (G1 and G2) and to examine effects of human *APOL1* renal risk allele and non-risk allele (G0) *in vivo*. Several approaches have been taken to reproduce APOL1-associated human disease in mouse models, including various expression systems, gene promoters, gene variants, mouse background strain, and interventions that impose kidney stress or damage. Here we review published work in this area and consider what is missing or incomplete and what possible future approaches might be taken.

With regards to generating or selecting a transgenic mouse model, it is important to consider aspects of transgene expression such as tissue specificity of expression and regulation of expression. There are many available transgene promoters and enhancers. The transgene may be inserted randomly or may be inserted into a selected genetic locus. Random insertion is simpler, but tissue expression may be influenced by proximity to strong enhancer elements; this potential problem can be avoided by adding locus control regions to the transgene, but at the cost of a larger and more complex transgene (15). Further, the genetic background of the mouse strain must be considered, as outcomes may vary with genetic background.

In 2016, Bruggeman et al. reported the first transgenic *APOL1* mouse model, using the mouse *Nphs1* promotor (16). Both APOL1-G0 and -G2 mice showed decreased podocyte density and preeclampsia. Using the mouse *Nphs1* promoter, which is highly specific within kidney for podocytes, allowed targeting of APOL1-transgene expression to the cell where APOL1 is expressed in human kidneys and from where it is thought to drive APOL1-mediated kidney disease. One limitation was that even though these investigators showed that APOL1 expression affected podocyte function, this effect was not specific to the renal risk alleles but also to common variant APOL1-G0.

Beckerman et al. took a different approach, using a Tet-on activatable overexpression system to generate APOL1 transgenic mice (17). They showed that mice with *Nphs1* promotor-driven APOL1-G1 and -G2 overexpression developed global and segmental glomerulosclerosis, compared with -G0 mice, which had no kidney phenotype. In individual mice, albuminuria levels correlated with *APOL1* expression levels. Mechanistically, reduced autophagy resulted in pyroptosis *i.e.*, inflammatory cell death. Similarly, Kumar and others used the Tet-On3G inducible system to investigate APOL1 functions and showed that A*POL1* risk alleles de-stabilized the junction complex, which contributes to cell-cell contact and cell-matrix adhesion (18). One limitation to all these transgenic mice is the difficulty in relating transgene expression levels to those seen in humans.

In another approach, Okamoto et al. studied human *APOL1* gene locus transgenic mice, where the transgene is a 47 kb human cDNA that contains sequence from chromosome 22, included within a bacterial artificial chromosome (BAC). This BAC contains the exons and intron of APOL1, as well as portions of APOL2 and MYH9. This approach facilitates studies of gene regulation using physiologic stimuli, such as interferon or agents that promote interferon expression (19). The APOL1-G2 variant mouse showed significantly higher albuminuria compared to APOL1-G0 mice, following exposure to a combination of interferon-γ (to stimulate APOL1 gene transcription), basic fibroblast growth factor, and puromycin aminonucleoside (to induce podocyte injury). Importantly, the study showed that APOL1-variant mRNA activated protein kinase R and that this activation contributed to the observed podocyte injury.

To replicate physiological *APOL1* gene regulation and expression levels as seen in humans, Aghajan et al. used 32 Mb fosmid DNA vectors in the generation of transgenic mice (20). The authors used interferon-γ injections to induce transient albuminuria, which was most prominent in APOL1-G1 mice. They demonstrated that pre-treatment with an anti-sense oligonucleotide targeting APOL1 could block the induction of albuminuria. This proof-of-concept study indicated that anti-sense oligonucleotides might have a therapeutic role in APOL1 nephropathy. However, many steps remain toward a clinical application of this technology.

McCarthy et al. recently reported the results of *APOL1* BAC transgenic mice that received *via* hydrodynamic tail vein injection an IFN-γ-expressing pCpGfree plasmid that lacks CpG motifs to achieve sustained IFN-γ levels in the animals. They showed robust induction of proteinuria and glomerulosclerosis in G1/G1 and G2/G2 mice but not in G0/G0 mice. They also showed that the heterozygous mice (G0/G1 or G0/G2) had greater proteinuria response than hemizygous mice (G1/- or G2/-), suggesting that APOL1-G0 does not rescue -G1 or -G2 allele toxicity. Further, mice with a multicopy G2 transgene (G2^multi^/G2^multi^) showed the greatest proteinuria response with worst prognosis, supporting the recessive nature of APOL1-nephropathy and the notion that disease is a function of the expression level of *APOL1* risk variant (21).

Ryu et al. used the BAC/APOL1 mouse model described above and showed that reduced cholesterol efflux and concomitant cholesterol accumulation may contribute to APOL1 nephropathy (22). In 2018, Kumar et al. generated a TetOn3G-APOL1 mouse model and identified a dual feedback loop in glomerular parietal epithelial cells, in which APOL1 suppresses miR-193a expression and miR-193a suppresses APOL1 expression (23).

Some models have involved dual-transgenic mice. Bruggeman et al. (24) studied the well-characterized Tg26 model of HIV-associated nephropathy, in which the presence of the six regulatory and accessory genes of HIV-1, under the control of viral long terminal repeats (LTR), develop FSGS and/or collapsing glomerulopathy (25). They reported that among Nphs1.APOL1 × Tg26 dual transgenic mice, APOL1-G0 × Tg26 mice showed less podocyte loss in compared with APOL1-G2 × Tg26 or Tg26 mice alone (24). These data suggest the intriguing idea that APOL-G0 protein might have some trophic or protective effect on podocytes, perhaps one that only manifests in certain injury settings.

Ge et al. studied a triple transgenic model, BAC/APOL1 × podocin-rtTA × TRE/NFATc1nuc mouse model (26). These mice manifest elevated levels of triglycerides and cholesterol in kidney, as well as glomerulosclerosis. The authors suggested that APOL1 risk variant expression increases the susceptibility to lipid-mediated podocyte injury, leading to mitochondrial dysfunction.

Another injury pathway was identified by Wakashin et al., who reported on a CAG-APOL1-B3 mouse model expressing the APOL1-B3 protein isoform under control of a CMV early enhancer/chicken β-actin promoter. The APOL1-B3 isoform, in contrast to the better characterized APOL1-A isoform, lacks a signal sequence for targeting to the secretory pathway and this results in cytosolic expression (27). These mice manifested podocyte injury and elevated IL-1β production for the G2 but not for the G0 variant. Further, APOL1-B3 interacted with NACHT, LRR, and PYD domains-containing protein 12 (NLRP12), a key regulator of toll-like receptor signaling. Through these pathways, APOL1-B3 and its risk variants seem to enhance inflammatory signaling in podocytes.

APOL1 is expressed most abundantly in liver, and this is the source of most plasma APOL1 (28). Targeted expression of APOL1 risk variants in murine livers by hydrodynamic gene delivery induced liver injury, demonstrating the cytotoxicity of the variants (11). Human data from kidney transplant studies suggest that only kidney-expressed, and not APOL1 expressed in other tissues has the potential to injure kidneys (29–31).

As shown above, mouse models have contributed in many ways to the understanding of molecular mechanisms of APOL1 kidney injury. Mouse kidney physiology shares many similarities to that of humans and diverse transgenic approaches are available, even though mice lack *APOL1*orthologs. Future research using existing and new mouse models will help to identify which mechanisms found *in vitro* are likely to be the relevant *in vivo* and relevant to human disease and may contribute to novel therapeutic approaches.

## Zebrafish

The zebrafish (*Danio rerio*) is the second most employed animal model in APOL1 research. Zebrafish express a protein with considerable homology to human APOL1 and this, together with its genetic tractability, has made it a useful model to study APOL1 function (32). Anderson et al. reported that translational suppression or CRISPR/Cas9 genome editing of *apol1* in zebrafish embryos results in podocyte loss and glomerular filtration defects (32). They also showed that complementation with the APOL1-G0 allele rescued the phenotype, but complementation with APOL-G1 or -G2 did not. Thus, the gene encoding APOL1 appears essential to glomerular function in fish, whereas this may not be the case in humans. A human subject who developed an unusual trypanosomal infection was found to be lacking in the *APOL1* gene but nevertheless had normal kidney function (10). However, the uniqueness of this intriguing finding warrants a cautious interpretation of the role of APOL1 or the lack of APOL1.

Kotb et al. confirmed that *apol1* knockdown causes podocyte damage in zebrafish (33). Olabisi et al. investigated zebrafish with G0, G1, or G2 *APOL1* variants, expressed using the Gal4-UAS system in podocytes or endothelial cells, using podocin and *Flk* (a receptor for vascular endothelial growth factor) promoters, respectively (34). Transgenic expression of *APOL1* G1 and G2, compared to the G0 allele, was associated with histologic abnormalities in zebrafish glomeruli but renal function remained normal. Bundy et al. investigated molecular pathways in zebrafish podocytes, using pathway analysis of differentially expressed transcripts, and showed enrichment for transcripts characterized by autophagy associated-terms, implicating autophagy pathways in APOL1 G2-associated kidney dysfunction (35).

The zebrafish model recapitulates certain aspects of molecular mechanisms acting downstream of human *APOL1* risk alleles, but the functional changes and the effects seen by knocking down APOL1 seem contradictory to observations in human subjects. Consequently, the specificities of zebrafish-APOL1 interactions have to be kept in mind and carefully further explored to advance our understanding of this animal model.

## Drosophila

*Drosophila melanogaster*, the common fruit fly, is a model system that offers ready access to genetic manipulation, enabling studies of protein function and protein-protein interaction. In 2017, two reports presented findings on effects of APOL1 expression on nephrocytes in *Drosophila* (36, 37). These models exploit structural and functional similarities of *Drosophila* pericardial nephrocytes to those of human podocytes and proximal tubular cells. The accessibility of *Drosophila* nephrocytes facilitates high resolution *in vivo* analysis of renal cells (36).

Fu et al. reported that ubiquitous expression of human *APOL1*-G0 or -G1 variants in *Drosophila* induced lethal phenotypes, with -G1 being more toxic than -G0. Expressing the *APOL1* transgene in nephrocytes impaired the acidification of organelles. Kruzel-Davila et al. reported that ubiquitous expression of the human APOL1-G1 and -G2 variants caused near-complete lethality in the flies, with no effect observed for -G0 (37). These effects are more severe than those seen in humans, perhaps due to higher expression levels in the flies or the particular pattern of tissue expression, but this study does lend support to a gain-of-dysfunction model for APOL1 variant cell toxicity. These authors also observed differential toxicity of the APOL1 risk alleles compared with non-risk alleles including disruption of endolysosomal processes.

The fly models have proven useful in showing a lethal phenotype and endolysosomal disruption by APOL1 risk alleles, and to identify interacting partners of APOL1. These models to address specific mechanisms that may be shared by nephrocytes and podocytes, but these two cell types have important differences that limit definitive conclusions.

## Discussion

Several factors should be considered when designing or selecting an animal model system that can most effectively and efficiently address the selected research questions.

### Promoter Selection

Promoter selection is of great importance, as it determines the specificity, kinetics, and dose of transgene expression. These parameters determine the types of questions that can be addressed. In the kidney, APOL1-mediated cytotoxicity is particularly prominent in podocytes. Consequently, many transgenic mouse studies have selected podocyte-specific promoters to interrogate podocyte-specific function of *APOL1*. However, APOL1 is not only expressed by podocytes but also expressed by renal endothelial cells (38). This finding warrants the application of endothelial cell-specific animal models to specifically investigate the effects of APOL1 on endothelial cells. It is true that APOL1 expression is still a controversial topic as Ma et al. reported the presence of APOL1 protein in kidney tubular cells suggesting a function of APOL1 there (39). Further, several studies have explored systemic expression of APOL1 to interrogate unbiased, systemic function of *APOL1*. Considering APOL1's ubiquitous expression in many organs plus its presence on HDL particles circulating in the bloodstream, systemic models offer avenues to uncover the remaining unknowns in APOL1 functions.

### Intervention and Expression System

Establishing a robust transgene phenotype is critical in generating a persuasive animal model. In human APOL1 nephropathy, APOL1 variant risk status appears to be insufficient for disease onset and a second factor, generally increased APOL1 expression, appears to be required. Interventions to increase transgene expression are also required in several APOL1 transgenic animal models to increase gene expression. IFN-γ activates the APOL1 promoter (19, 20) and doxycycline activates the Tet-On promoter (17, 18). Puromycin aminonucleoside and/or basic fibroblast growth factor have been used to induce podocyte injury (19). Identifying effective interventions that result in overt phenotypes remain challenging.

### Background Strain of Mouse Models

Mouse background strain can have a substantial effect on susceptibility to disease or response to a disease-promoting intervention. A common background strains for transgenic mice is FVB/N, due to the relative ease of injecting the large pronuclei in the zygote. This strain has also proven very useful in kidney studies, as other strains including strains 129S1 or B6 turned out to be more resistant to kidney injury.

### Findings From Animal Models

**Figure 1:**
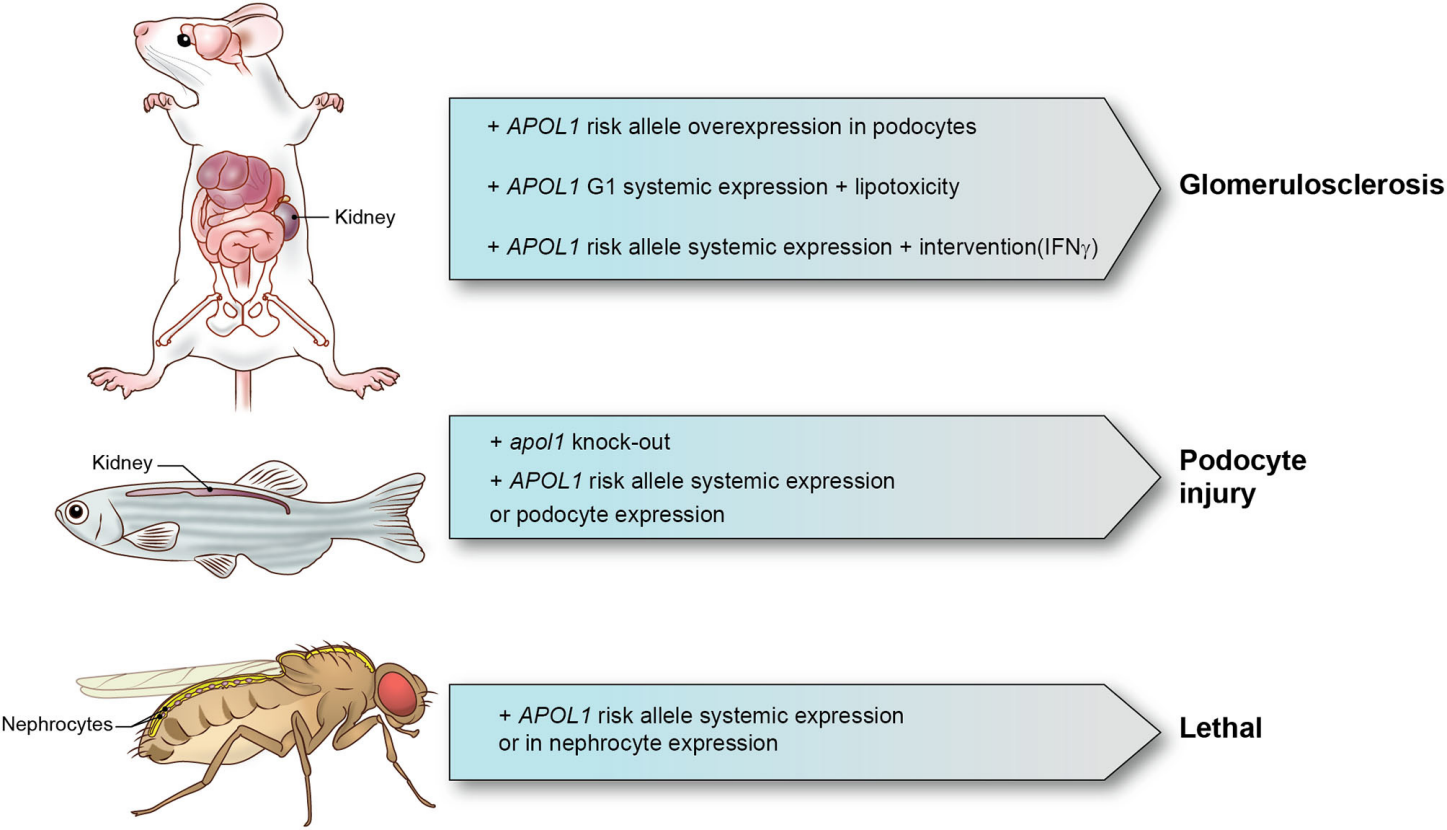
APOL1 animal models and phenotypes. Representative APOL1 animal models with interventions and phenotypes are shown.

In Table 1, we summarize the models that have been reported. Although a range of mechanisms have been documented for *in vitro* models that investigate APOL1 function, only limited number of mechanisms have been identified and confirmed in animal models. We also summarized major animal models in Figure 1. Animal models come with a variety of limitations. Designing and generating an animal model for complex human genetic diseases is challenging especially if specific physiology or environmental factors are contributing elements. The APOL gene family arose in primates and diversified rapidly, by gene duplication, on the one hand, and by gene loss and pseudogenization on the other hand. This gene family is absent from all the commonly used research animal species, including mice. This raised the potential concern that other molecules that co-evolved in primates to interact with APOL1 and perhaps regulate or alter its activity would be missing in mice and other laboratory animals. Although modern molecular biology methods allow a gene or genomic fragments containing gene and extended flanking regions to be introduced in these model systems, it remains a challenge to decipher how the transgene expression affects the transgenic animal and how these effects relate to pathological processes and especially their kinetics observed in human subjects. The duty remains to carefully explore how far a model can inform on the human setting.

**Table 1:** Summary of APOL1 animal models.

**Species**	**Transgene**	**Variants**	**Cell**	**Method**	**Findings**	**Phenotype**	**Ref**
Mouse	Nphs1.rTA × TRE-APOL1	G0, G1, G2	Podocytes	Tet-on	↑APOL1 expression, ↓autophagy, ↑pyroptosis	Glomerulosclerosis in G1 and G2	(17)
	Pax8.rTA × TRE-APOL1	G0, G1, G2	Proximal tubules	Tet-on	No	No kidney disease	(17)
	TetOn3G-APOL1	G0, G1, G2	Podocytes	Tet-on	De-stabilizes the adherens complex	Albuminuria in G1/G1 and G1/G2	(18, 23)
	Nphs1.APOL1	G0, G2	Podocytes		No change in necrosis, apoptosis, autophagy	↓podocyte density, Preeclampsia in G0 and G2	(16)
	BAC (Merck)	G0, G1, G2	Native	IFN-*g*/PAN/FGF	APOL1 mRNA activates PKR	Albuminuria in G2	(19)
	Fosmid (Ionis)	G0, G1, G2	Native	IFN-γ	ASO blocks APOL1 expression	Albuminuria in G1	(20)
	BAC	G0, G1, G2	Native	pCpG-Muγ	Phenotype severity: G2^multi^/G2^multi^>G2/G2 >G2/G0>G2/-	Glomerulosclerosis in G1/G1 and G2/G2	(21)
	CAG-APOL1-B3	G0, G2	Systemic	uninephrectomy	↑IL-1β production	Albuminuria in G2	(27)
	TetOn3G-APOL1 × Tg26	G0, G1, G2	Podocytes	Tet-on	Not stated	Not stated	(23)
	Nphs1.APOL1 × Tg26	G0, G2	Podocytes		↑Podocyte in G0	No change	(25)
	BAC-APOL1 × Podocin-rtTA × NFATc1nuc	G0, G1	Podocytes	Tet-on	↑TG and Chol in G1	FSGS in G1	(22)
	pRG977-APOL1	G0, G1, G2	Liver-dominant	hydro- dynamic gene delivery	giant cell formation, macrophage infiltration: G1 > G2	severe liver necrosis, calcification: G1 > G2	(11)
Zebrafish	KO-zebrafish-apol1	G0, G1, G2	Systemic	Morpholino oligonucleotide- knockdown, CRISPR/Cas9	podocyte loss and glomerular filtration defects in KO, rescued by G0 only	Edema in KO	(32)
	KO-zebrafish-apol1	G0	Systemic	Morpholino oligonucleotide-knockdown	leakage of the filtration barrier in KO	Edema in KO	(33)
	APOL1	G0, G1, G2	Podocyte or endothelial cell	UAS-Gal4 system	Segmental podocyte FP effacement and irregularities in podocyte-G1	No dysfunction	(34)
	APOL1	G0, G2	Podocyte or endothelial cell	mRNA microinjection into the yolk	autophagy pathways upregulated in G2 podocytes	Not stated	(35)
*Drosophila*	APOL1	G0, G1	Systemic or nephrocyte	UAS-Gal4 system	impaired the acidification of organelles	Lethal in G0 and G1; G1 > G0	(36)
	APOL1	G0, G1, G2	Systemic or nephrocyte or eye-specific	UAS-Gal4 system	disruption of the endolysosomal processes	Lethal in G1 and G2	(37)

*Shown are 18 transgenic models of APOL1 pathophysiology, involving three animal species. Methods used to generate models are shown.*

*BAC, bacterial artificial chromosome; KO, knock out; CAG, cytomegalovirus immediate early enhancer-chicken beta-actin hybrid promoter; FGF, fibroblast growth factor; PAN, puromycin aminonucleoside; rtTA, reverse tetracycline transactivator; TRE, tet-response element; UAS-GAL4, upstream activation sequence, which responds when GAL4 is expressed and activates the target gene; pCpG-Muγ, an IFN-γ-expressing pCpGfree plasmid that lacks CpG motifs*.

### Future Directions for Animal Model Research

In patients, APOL1 kidney disease appears to follow a two-hit model, whereby a genetic predisposition (presence of two *APOL1* kidney risk alleles) is coupled with factors that increase *APOL1* gene expression (interferon being the most well-documented). Given an increase in *APOL1* gene expression, it remains unclear to what extent peak magnitude of expression vs. area under the curve of expression is more important in inducing glomerular injury. Also, there may be factors other than interferon that increase gene expression or alternatively synergize with what might otherwise be sub-pathogenic APOL1 expression levels to induce kidney injury. Future animal studies will be required to understand the relationship between the kinetics of gene expression and glomerular injury.

Animal studies offer the opportunity to examine what other factors, beyond APOL1 variant expression level, might alter the timing and degree of glomerular injury. These might include age, sex, body weight and various co-morbidities. Single cell methods that provide unprecedented insights into otherwise not recognizable changes in per-cell based transcriptomes may also shed light into molecular mechanisms by APOL1, together with novel animal models. Further APOL1 animal studies will be warranted to better understand to which factors have to be paid more attention in the population carrying *APOL1* risk alleles.

## Author Contributions

TY reviewed the literature and drafted the manuscript. KL and JK provided comments. TY, JH, and JK edited the manuscript. All authors read and approved the final draft.

## Funding

This work was supported by the NDDK Intramural Research Program, NIH, Bethesda, MD.

## Conflict of Interest

The authors declare that the research was conducted in the absence of any commercial or financial relationships that could be construed as a potential conflict of interest.

## Publisher's Note

All claims expressed in this article are solely those of the authors and do not necessarily represent those of their affiliated organizations, or those of the publisher, the editors and the reviewers. Any product that may be evaluated in this article, or claim that may be made by its manufacturer, is not guaranteed or endorsed by the publisher.
